# Accuracy of C-reactive protein and a differentiated white cell count in diagnosing tuberculosis

**DOI:** 10.4102/sajid.v38i1.481

**Published:** 2023-05-29

**Authors:** Gideon Ruder, Richard M.N. Carter, Gina Joubert

**Affiliations:** 1 Department of Internal Medicine, Faculty of Health Sciences, University of the Free State, Bloemfontein, South Africa; 2 Department of Biostatistics, Faculty of Health Sciences, University of the Free State, Bloemfontein, South Africa

**Keywords:** C-reactive protein, empiric tuberculosis treatment, neutrophil-lymphocyte ratio, HIV-positive people, smear-negative tuberculosis, South Africa, white cell count

## Abstract

**Background:**

Tuberculosis (TB) is treatable with a high cure rate. In South Africa, 70% of pulmonary TB is microbiologically confirmed. Autopsy studies of HIV-positive people found 45.7% undiagnosed TB cases.

**Objectives:**

The primary objective investigated whether CRP and a differentiated white cell count (WCC) and ratios thereof are useful screening tools for TB.

**Method:**

This retrospective cross-sectional study included adult patients admitted to two tertiary hospitals in Bloemfontein with TB workups between April 2016 and September 2019. National Health Laboratory Service (NHLS) provided laboratory data. Tuberculosis Xpert^®^ MTB/RIF, Xpert^®^ MTB/RIF Ultra and TB culture were used as reference standard for TB diagnosis.

**Results:**

The study population comprised 1294 patients; 15.1% had TB, 56.0% were male and 63.1% HIV-positive. Patients with TB were younger (*p* < 0.0001; 95% CI: –8;–3 years). In the total population, WCC had the highest area under the curve (0.59). White cell count (*p* < 0.0001), neutrophils (*p* = 0.0003) and lymphocytes (*p* = 0.0394) were lower in TB patients, and CRP-WCC ratio (CWR) (*p* = 0.0009) and CRP-lymphocyte ratio (CLR) (*p* = 0.0386) higher. In HIV-positive patients, WCC (*p* = 0.0003), neutrophils (*p* = 0.002) and lymphocytes (*p* = 0.0491) were lower in TB patients and CWR (*p* = 0.0043) higher. No parameter reached the World Health Organization screening targets of 70% specificity with 90% sensitivity.

**Conclusion:**

Differentiated WCC and CRP are not useful in screening hospitalised patients for TB in our setting.

**Contribution:**

Our study guides future research to augment current screening and diagnostic algorithms for TB, specifically in advanced HIV disease.

## Introduction

Tuberculosis (TB) is a treatable condition with a cure rate of 80.7% in the Mangaung Metropolitan Municipality, Free State. Yet, it was the largest contributor to death in South Africa (SA) before 2020.^1,2^ Only 70% of pulmonary TB is microbiologically confirmed in SA and 55% globally.^3,4^ Autopsy studies of HIV-positive people indicated that 45.7% of TB cases had been undiagnosed despite high rates of empiric TB treatment.^5^ Undiagnosed individuals continue the spread of disease in communities and care facilities.^6,7,8,9^ Overdiagnosis is also hazardous – TB treatment can be toxic, with serious adverse events reported in 26.7% of HIV-positive and 13.3% of HIV-negative patients.^10^

The World Health Organization (WHO) considers the association between HIV and TB as the main impediment to TB control in HIV-prevalent settings.^11,12,13^ Underlying reasons include HIV complicating TB diagnosis with poorer comparative test sensitivity, increased paucibacillary disease, extra-pulmonary TB and delays in sample collection.^13,14,15,16^ This deadly syndemic has its epicentre rooted in southern Africa.^4^

The tools currently available for microbiological confirmation of TB in the South African context include Xpert^®^ MTB/RIF Ultra (hereafter referred to as Ultra) – previously TB Xpert^®^ MTB/RIF (hereafter referred to as Xpert), TB culture and lateral flow urine lipoarabinomannan (LAM) assay. The swathe of non-microbiologically confirmed TB can be partially explained by the deficits in the diagnostic armamentarium: delays in sample collection (Ultra and TB culture), prolonged processing time (TB culture), poor sensitivity in paucibacillary extra-pulmonary disease (Ultra and culture) and limited indications with poor sensitivity (LAM assay).^17,18,19,20^

Cytokine production and acute-phase responses differ among various conditions. C-reactive protein (CRP) is a non-specific acute phase reactant that correlates with the severity of systemic inflammation.^21^ Raised neutrophil and lowered lymphocyte counts are typical of bacterial infection.^19,22,23^ The immune response to TB depends on cellular immunity and utilises T-helper lymphocytes to form granulomas.^13,24,25^ A preponderance of lymphocytes in TB infection has been described; however, HIV is known to cause CD4 T lymphocyte depletion and dysfunction.^13,26^

C-reactive protein and white cell count (WCC) differential have some utility in diagnosing various infections.^21,27,28,29,30,31,32,33^ In the South African context, there are some data for TB diagnosis: CRP has been posited as a screening tool for TB in asymptomatic HIV-positive people, with normal values making TB unlikely. C-reactive protein utility in diagnosing TB in seriously ill hospitalised HIV-positive people with respiratory illnesses and in symptomatic outpatients is limited to clinical prediction rules.^34,35,36^ White cell count independently predicts TB in seriously ill hospitalised South African HIV-positive people.^37^ International data have shown some encouraging although not uniform results for the utility of neutrophil-lymphocyte ratio (NLR), WCC and CRP in TB diagnosis.^23,33,38,39,40,41,42^

To the authors’ knowledge, no data on NLR as part of TB diagnostics in HIV-endemic settings or ratios of CRP to a differentiated WCC have been published. Sensitivity and specificity of 80% for these biomarkers to predict TB compared to other bacterial causes of community-acquired pneumonia were anticipated, lower than the quoted literature^23,38^ because of the unknown effect of HIV.

The primary objective was to investigate whether CRP and a differentiated WCC (and ratios thereof) are useful screening tools to distinguish TB from other illnesses in our setting. Secondary objectives were to describe the CRP and differential WCC of TB patients in our setting and to analyse according to HIV status.

## Methods

This was a retrospective cross-sectional study of participants’ laboratory data, presented according to the Standards for Reporting of Diagnostic Accuracy Studies (STARD) guidelines (checklist Appendix 1).^43^ Inclusion in the study required admission to either of the two tertiary-level state hospitals in Bloemfontein, with a TB workup done during the admission. A TB workup and the reference standard for this study are defined as a TB Xpert, Ultra or TB culture performed on fluid or tissue and done and interpreted according to the manufacturer’s instructions.^44^

Tuberculosis culture is performed and interpreted using the MGIT 960 liquid culture system (BD Diagnostics, United States).^45^ The index tests were CRP and differentiated WCC. C-reactive protein was measured by Cobas 6000 c-501 (Roche Diagnostics GmbH, Mannheim, Germany).^46^ Differentiated WCC was measured by Advia 2120i blood cell analyser (Siemens Healthcare Diagnostics Inc., Tarrytown, New York, United States).^47^

Exclusion criteria included:

- age younger than 18 years
- less than two of the following tests performed: CRP, WCC, neutrophil, lymphocyte
- TB tests with indeterminate results and
- tests ordered from oncology wards (malignancies and treatment could markedly interfere with test results).

Both hospitals sent samples for the relevant laboratory tests to the National Health Laboratory Service (NHLS). Data retrieval from NHLS was done via the Academic Affairs and Research Management System (AARMS). The NHLS stores data for approximately 5 years. The data request was approved by NHLS in September 2020. Data were requested chronologically for the approved period (before October 2019) for qualifying patients until data were obtained for a minimum of 300 patients with and 300 patients without TB (or up to the limit of the NHLS data archive), to ensure narrow confidence intervals (CI) (exact timeframe April 2016 – September 2019).

The NHLS extracted, cleaned and anonymised the data. Data included CRP, WCC, neutrophil count, lymphocyte count, age, gender (as per patient registration at the hospital and subsequently NHLS) and HIV data, including CD4 and HIV viral load. Data for sex (female, male, unknown, none of the above), Xpert or Ultra (negative, positive), TB culture (negative, positive), HIV status (negative, positive), viral load (suppressed, unsuppressed – detectable) and CD4 count (< 100 cells/µL, < 200 cells/µL, < 350 cells/µL and ≥ 350 cells/µL) were requested as categorical variables. The other results were received as numerical variables.

Admission status was determined by NHLS records that state the location from which the tests were done. Timeframes for the inclusion of test results were selected to ensure the studied blood results accurately reflect the likely clinical status of the patient at the time of the TB workup.

### Data cleaning

Results from the CRP tests and differentiated WCC performed, within a 1-week window before and 1 day after TB tests (further described as a research incident [RI]), were collected. If several CRP test results and differentiated WCC were found in a single RI, the initial results in the sequence were used.

Only one RI from a 2-month window was included and measured from the date of the included TB test. Any positive test in the TB workup was considered TB, and the first positive test was considered as the RI. If all tests were negative, the outcome was considered negative with the first test included as the RI.

HIV status was determined by HIV-related tests (HIV enzyme-linked immunosorbent assay [ELISA], HIV polymerase chain reaction [PCR], HIV viral load and CD4 count) ordered on the patient before the RI and 3 months thereafter. If any of these diagnostic tests was positive, the patient was considered HIV positive. If an HIV viral load or CD4 count was found to have been done by NHLS during the stipulated timeframe, it was assumed the patient is HIV positive. Those neither HIV positive nor HIV negative were considered HIV unknown. The CD4 count and HIV viral load nearest the RI within a 6-month window of the RI were included.

### Pilot study

A pilot study was conducted, in which data of the first 10 patients on the list provided by the NHLS were analysed. As no problems arose, these cases were incorporated into analyses.

### Statistical analysis

Analysis was performed by the university’s Department of Biostatistics, using SAS Version 9.4. Categorical variables were summarised by frequencies and percentages and numerical variables by medians and interquartile ranges (IQR) because of skew distributions. Mann–Whitney tests with 95% CIs for median differences were performed to compare patients with TB to those without TB regarding numerical variables. Logistic regression of each variable compared to the TB diagnosis was performed to determine sensitivities, specificities, positive predictive and negative predictive values, likelihood ratios and areas under the curve (AUC). All variables with significant difference between TB and non-TB patients were entered into a logistic regression model with backward elimination to identify variables jointly significantly associated with TB diagnosis. The above analyses were stratified by HIV status. Missing laboratory values for a specific variable were excluded from analysis of that variable, and the numbers analysed were stated throughout.

### Ethical considerations

Approval to perform this study was obtained from the University of the Free State Health Sciences Research Ethics Committee (ethics number UFS-HSD2019/1468/2801), Free State Department of Health and the NHLS Academic Affairs and Research office. This article did not contain any studies involving human participants performed by any of the authors.

## Results

**FIGURE 1:**
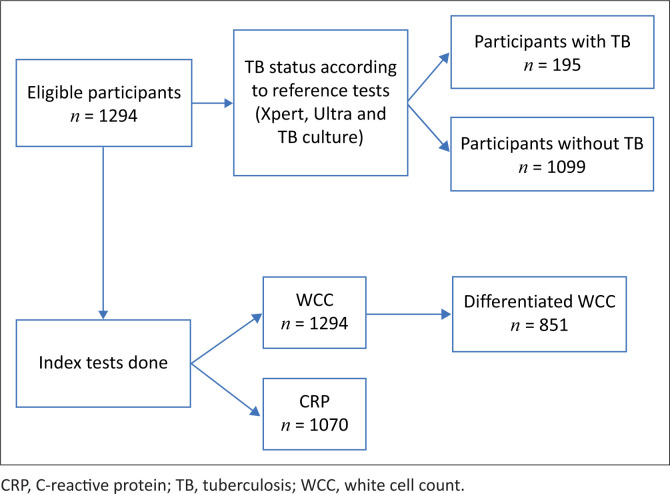
Flowchart of patients included.

The NHLS provided cleaned data, in which the total study population had a WCC (*n* = 1294), 1070 had a CRP result and 851 had a WCC differential captured. Data regarding the number of patients for whom TB workup had been done, but with insufficient blood results for inclusion were not provided. Figure 1 demonstrates the flow of patients included.

The patients were predominantly male (56.0%). Those with TB were younger than those without TB (Table 1). Of the 1294 patients, 195 (15.1%) had TB. Of the 195 participants with TB, 64 (32.8%) were diagnosed by culture. The TB positivity rate was similar among males and females (15.9% and 14.0%, respectively).

**TABLE 1:** Unadjusted clinical and laboratory characteristics of the total study population investigated for tuberculosis.

Variable	TB patients			Non-TB patients			*p*	95% CI for median difference (TB minus non-TB)
	*n*	Median	IQR	*n*	Median	IQR		
Age	195	38	29–50	1099	43	33–59	< 0.0001	−8; −3
CRP	153	122	53–187	917	106	31–215	0.2012	−5; 25
WCC	195	8.05	5.21–10.78	1099	9.5	6.29–13.87	< 0.0001	−2.31; −0.82
N	195	5.67	3.28–8.67	656	7	4.23–10.75	0.0003	−1.92; −0.58
L	195	0.98	0.57–1.65	656	1.15	0.72–1.62	0.0394	−0.22; −0.01
NLR	195	5.69	3.07–10.97	656	6.27	3.44–11.25	0.4100	−1.06; 0.43
CWR	153	13.25	5.98–22.83	917	9.64	3.82–19.03	0.0009	1.10; 4.67
WLR	195	7.71	4.65–14.19	656	8.37	5.18–14.27	0.3848	−1.26; 0.46
CLR	153	133.05	43.48–271.54	474	97.13	28.91–211.54	0.0386	0.42; 34.89

CI, confidence interval; CLR, CRP-lymphocyte ratio; CRP, C-reactive protein in mg/L; CWR, CRP-WCC ratio; IQR, interquartile range; L, lymphocyte in × 10^9^/L; N, neutrophil in × 10^9^/L; NLR, neutrophil-lymphocyte ratio; TB, tuberculosis; WCC, white cell count in × 10^9^/L; WLR, WCC-lymphocyte ratio.

In the total study population, WCC and neutrophils were significantly lower, and CRP-WCC ratio (CWR) and CRP-lymphocyte ratio (CLR) significantly higher in patients with TB than those without TB (Table 1). The maximum calculated AUC for any of these statistically significant variables was for WCC (0.59). A WCC between 8.05 × 10^9^/L and 9.49 × 10^9^/L had sensitivities and specificities ranging between 50% and 65%, but no cut-off value had a sensitivity and specificity above 60%. When including all individually statistically significant variables in a logistic regression model, the only variable retained as significant was the CWR with AUC 0.58. The sensitivity of NLR of < 7 for diagnosis of TB was 57.4% (95% CI: 50.2%; 64.4%), and the specificity was 45.1% (95% CI: 41.3%; 49.0%).

Of those patients with a CRP value < 10 mg/L, 10.6% had TB (9/85). A CRP value > 10 mg/L had a sensitivity of 94.1% (95% CI: 88.8%; 97.1%) and specificity 8.3% (95% CI: 6.6%; 10.3%), respectively, and a negative likelihood ratio of 0.7 to diagnose TB.

Data for HIV-positive patients are provided in Table 2. Of 817 patients with HIV (63.1% of the sample), 132 (16.2%) had TB.

**TABLE 2:** Unadjusted clinical and laboratory characteristics in HIV-positive patients investigated for tuberculosis.

Variable	TB patients			Non-TB patients			*p*	95% CI for median difference (TB minus non-TB)
	*n*	Median	IQR	*n*	Median	IQR		
Age	132	38	29.5–49	685	42	33–57	0.0015	−7; −2
CRP	101	131	62–189	576	116.5	39–216.5	0.3702	−11; 29
WCC	132	7.68	5.03–10.48	685	9.43	6.38–13.68	0.0003	−2.63; −0.82
N	132	5.27	3.04–8.61	407	7.01	4.29–10.54	0.0020	−2.12; −0.49
L	132	0.97	0.53–1.58	407	1.15	0.69–1.62	0.0491	−0.27; 0
NLR	132	5.76	3.04–11.86	407	6.24	3.21–11.87	0.6705	−1.14; 0.72
CWR	101	15.2	6.61–24.98	576	10.74	4.11–20.11	0.0043	0.96; 5.61
WLR	132	8.27	4.72–14.23	407	8.36	5.06–14.36	0.5857	−1.39; 0.74
CLR	101	131.33	52.94–285.96	298	110.94	31.91– 256.32	0.1806	−7.54; 46.12

CI, confidence interval; CLR, CRP-lymphocyte ratio; CRP, C-reactive protein in mg/L; CWR, CRP-WCC ratio; IQR, interquartile range; L, lymphocyte in × 10^9^/L; N, neutrophil in × 10^9^/L; NLR, neutrophil-lymphocyte ratio; TB, tuberculosis; WCC, white cell count in × 10^9^/L; WLR, WCC-lymphocyte ratio.

Among HIV-positive patients, WCC, neutrophils and CWR showed statistically significant differences between those with and without TB (Table 2). The maximum calculated AUC was for WCC (0.60). When including all individually statistically significant variables in a logistic regression model, the only variable retained as significant was the WCC. The sensitivity and specificity at NLR of < 7 for the diagnosis of TB were 56.8% (95% CI: 47.9%; 65.3%) and 46.2% (95% CI: 41.3%; 51.2%), respectively.

Of those HIV-positive patients with a CRP value < 10 mg/L, 10.6% had TB (5/47). A CRP value > 10 mg/L had a sensitivity of 95.1% (95% CI: 88.3%; 98.2%) and specificity of 7.3% (95% CI: 5.4%; 9.8%), with a negative likelihood ratio of 0.7 to diagnose TB.

Data for the HIV-negative patients are provided in Table 3. Of 226 (17.5%) HIV-negative patients, 32 (14.2%) had TB. Those with TB were younger. No laboratory characteristic showed any statistically significant difference between patients with and without TB. The HIV status of 251 (19.4%) patients was unknown.

**TABLE 3:** Unadjusted clinical and laboratory characteristics in HIV-negative patients investigated for tuberculosis.

Variable	TB patients			Non-TB patients			*p*	95% CI for median difference (TB minus non-TB)
	*n*	Median	IQR	*n*	Median	IQR		
Age	32	33.5	25–51.5	194	48	30–57	0.0139	−15; −1
CRP	26	112	47–237	166	75.5	21–186	0.1403	−9; 55
WCC	32	8.97	4.36–11.43	194	10.05	6.15–13.92	0.1507	−3.39; 0.58
N	32	6.24	3.15–8.77	112	7.87	4.27–11.67	0.0675	−3.38; 0.17
L	32	0.94	0.66–1.76	112	1.14	0.8–1.58	0.7042	−0.32; 0.25
NLR	32	5.04	3.02–11.97	112	5.77	3.66–11.12	0.3414	−2.53; 0.90
CWR	26	10.59	4.59–22.46	166	7.84	2.76–15.27	0.0751	−0.42; 7.19
WLR	32	7.05	4.5–14.22	112	8.11	5.56–13.13	0.4335	−2.61; 1.29
CLR	26	119.62	28–232.17	84	86.08	29.47–157.95	0.2658	−18.06; 84.02

CI, confidence interval; CLR, CRP-lymphocyte ratio; CRP, C-reactive protein in mg/L; CWR, CRP-WCC ratio; IQR, interquartile range; L, lymphocyte in × 10^9^/L; N, neutrophil in × 10^9^/L; NLR, neutrophil-lymphocyte ratio; TB, tuberculosis; WCC, white cell count in × 10^9^/L; WLR, WCC-lymphocyte ratio.

Cut-off values for 90% sensitivity and 70% specificity for the diagnosis of TB are displayed in Table 4 for all laboratory parameters. These values are based on the WHO’s pre-defined ideal sensitivity and specificity cut-offs for screening tools for TB.^48^ In the total study population, the parameter with the best sensitivity with 70% specificity is WCC at 44% (value of 7.04 × 10^9^/L). In the total study population, the parameter with the best specificity with 90% sensitivity is CWR at 17.8% (value of 2.66 × 10^9^/L). In HIV-positive patients, the parameter with the best sensitivity with 70% specificity is WCC at 44.7% (value of 7.1 × 10^9^/L). In HIV-positive patients, the parameter with the best specificity with 90% sensitivity is CWR at 18.1% (value of 2.96 × 10^9^/L).

**TABLE 4:** Ninety percent sensitivity and 70% specificity cut-offs and corresponding values for the diagnosis of tuberculosis in the total study population and HIV-positive patients investigated for tuberculosis.

Variable	Total study population				HIV-positive patients			
	Sensitivity at 70% specificity (%)	Value	Specificity at 90% sensitivity (%)	Value	Sensitivity at 70% specificity (%)	Value	Specificity at 90% sensitivity (%)	Value
CRP	23.5	192	17.0	20	24.8	196	16.8	22
WCC	44.0	7.04	12.9	17.77	44.7	7.1	10.4	18.48
N	42.1	4.72	11.7	15.1	43.2	4.72	9.6	15.67
L	39.0	0.77	11.3	2.1	37.9	0.75	11.6	2.07
NLR	37.4	3.74	9.0	19.79	34.1	3.71	10.1	19.87
CWR	37.9	17.01	17.8	2.66	41.5	18.03	18.1	2.96
WLR	38.0	5.85	9.5	22.35	36.4	5.78	11.3	22.66
CLR	34.0	187.04	15.0	13.68	38.6	68.14	15.1	15.89

CLR, CRP-lymphocyte ratio; CRP, C-reactive protein in mg/L; CWR, CRP-WCC ratio; L, lymphocyte in × 10^9^/L; N, neutrophil in × 10^9^/L; NLR, neutrophil-lymphocyte ratio; TB, tuberculosis; WCC, white cell count in × 10^9^/L; WLR, WCC-lymphocyte ratio.

Of the 817 HIV-positive patients, 233 (28.5%) had HIV viral loads measured, of which 135 (57.9%) were suppressed. Of 340 HIV-positive patients (41.6%) with CD4 counts, 108 (31.8%) had < 100 cells/µL; 78 (22.9%) < 200 cells/µL; 66 (19.4%) < 350 cells/µL and 88 (25.0%) had ≥ 350 cells/µL.

## Discussion

Our data show that CRP and WCC differential are not useful tools when used in isolation or as ratios in diagnosing or excluding TB in hospitalised (tertiary hospitals) patients in our high-burden TB and HIV setting. This was the case for the total study population (maximum AUC of 0.59) and HIV-positive patients (maximum AUC 0.6). When considering the WHO’s pre-defined minimum sensitivity and specificity (90% and 70%, respectively), the data clearly illustrate the inability of these parameters as screening tools for TB.^48^

Strengths of our study include a large sample size (*n* = 1294) and many HIV-positive patients (*n* = 817, 63.1%), reflecting the burden of disease in our setting. The patients in this study were not limited to those being worked up for pulmonary TB. We collected data from various in-hospital settings. The retrospective design allowed us to collect real-world data and negate potential biases of our index tests on clinicians’ decision-making. Our data were from the pre-coronavirus disease 2019 (COVID-19) era, suggesting relative stability in the diagnostic milieu. Head office AARMS staff cleaned and anonymised the data.

Our limitations included an inability to reach our initial target of 300 patients with and 300 without TB because of a long delay between ethical approval and approval of the data request – leaving limited time for archived data. However, it is unlikely that additional patients would have altered the results significantly. Our patients were limited to those in tertiary-level hospitals. The patient population of a tertiary hospital might not be generalisable to other levels of hospital care because of the potential attention of other healthcare workers within the referral system before arrival at the tertiary hospital. By only including hospitalised patients, we sought to simulate a symptomatic study population as part of our primary objectives to ‘distinguish TB from other illnesses’, yet we acknowledge that many admissions to tertiary hospitals are elective visits for patients who were not ill at the time of their admission.

The retrospective study design limited our insight into the appropriateness of the TB workup, the patients’ comorbid disease profile, admission diagnosis and therapy provided. Limited CRP and WCC results were considered in data analysis to negate the influence of therapy bias. Valid arguments could be made to exclude additional patient groups (elderly, critically ill, etc.). Further exclusions based on site of admission, with the heterogeneity of diagnoses in settings compared to oncology wards, would have severely compromised patient numbers. By including multidisciplinary facilities, such as the intensive care unit (ICU), oncology patients may have inadvertently been included in our study. The large sample size could potentially have negated this. Information was not available regarding the availability of clinical data and other laboratory results to the NHLS staff performing the initial tests or the total number of patients with positive TB culture, Xpert or Ultra results because of a limitation in the data request to NHLS. This data might influence our study’s utility for future research but not the outcomes of our results. Not all clinicians routinely do CRP tests and WCC and differential count, which can confer selection bias. Determination of HIV status relied on a combination of absolute and surrogate markers (HIV viral load and CD4 count testing). It would have been preferable to have confirmed HIV status via direct means (confirmatory testing per protocol or history) as opposed to the surrogates used. This was not feasible because of the retrospective nature and the numbers required. Because of the large amount of missing data, further sub-analyses regarding CD4 counts and viral loads were not done.

Some patients in our study may have had TB diagnosed by other means – histology, urine LAM, clinical decision rules, empiric diagnosis – and featured as TB negative in our study. This limitation is mirrored in other studies.^35,36,37^ The reference standard for TB diagnosis is mycobacterial culture.^12^ It is, however, an imperfect gold standard because of the predilection for sampling errors and technical variation.^49,50,51^ The use of Xpert as an initial TB diagnostic test was formally included into South African guidelines in 2014.^17^ Although widely used, Xpert and Ultra have limited sensitivity. Xpert or Ultra can remain positive after the resolution of TB although the WHO does not consider these false-positive cases overly concerning.^15^

The finding that WCC was the most useful parameter from our data was unsurprising in the context of a prospective study published by Griesel et al.^37^ that included seriously ill HIV-positive patients admitted to regional hospitals in South Africa. They found a decrease in the odds ratio for TB of 0.9 (95% CI: 0.87; 0.93) for every WCC increase by 1 × 10^9^/L, and high WCC shows a good negative predictive value for TB. White cell count was included in their clinical prediction rule. From our data, however, signs of utility were less encouraging. Median values of WCC of patients with and without TB were within the normal reference range of 3.9 × 10^9^/L to 12.6 × 10^9^/L (8.05 × 10^9^/L and 9.5 × 10^9^/L, respectively).^52^

A systematic review and meta-analysis showed utility in screening people (including HIV-positive people) for TB with CRP in the outpatient setting with a cut-point of 10 mg/L, having a pooled sensitivity and specificity of 93% (95% CI: 85%; 97%) and 62% (95% CI: 42%; 79%), respectively.^34^ This high sensitivity is mirrored in data of symptomatic South African HIV-positive people.^35^ From the above review, specificity is disappointing in both outpatients presenting with symptoms as well as inpatients (26% [95% CI: 19%; 34%] and 21% [95% CI: 6%; 52%], respectively).^36^ C-reactive protein alone did not aid in diagnosing ill outpatients or inpatients.^35,36^ C-reactive protein has some diagnostic utility within a clinical prediction rule in symptomatic outpatients in South Africa.^36^ In our data, 10.6% of patients with a CRP value < 10 mg/L had TB, and the specificity of CRP > 10 mg/L for TB diagnosis was 8.3% (95% CI: 6.6%; 10.3%).

We did not reproduce encouraging results on NLR from South Korean and Egyptian studies of hospitalised patients reporting AUCs above 0.90.^38,53^ There are several potential reasons for the discrepancy: HIV was not considered and is uncommon in both countries.^54,55,56,57^ Neutrophil counts in those without TB were significantly lower in our data (HIV-positive and HIV-negative patients) than the other studies. However, patients with TB were not compared directly to those with respiratory infections. Neutrophil precursors were not included in our neutrophil count. It is unclear whether they were included in other data. To negate this, we studied ratios of all the variables – which still did not yield clinically significant cut-offs. Lymphocyte counts in those with TB were lower in our data (HIV-positive and HIV-negative patients) compared to others. Differences in racial groups, although not specifically studied, were expected between our South African-based, South Korean and Egyptian studies. Variations in WCC reference indices among racial groups are established.^58^ Participants in the Korean study were significantly older – the median ages in those with TB and community-acquired pneumonia were 54 years (range 20–83 years) and 70 years (range 18–86 years), respectively.

Our data answer some pressing questions and guides further exploration of the topic. Patients under investigation for TB can have a wide range of CRP and WCC values. Therefore, TB cannot be diagnosed or excluded solely on CRP and WCC differential in symptomatic persons in our setting. These parameters should only be used in validated diagnostic algorithms.^36^ Our research re-emphasises the need to expeditiously collect high-quality and appropriate samples for TB diagnostic testing.

In the WHO Advanced HIV Disease (AHD) guidelines, research gaps for further TB screening and diagnostic algorithms (specifically in AHD) were identified, with various non-microbiological laboratory and clinical parameters being considered.^59^ Empiric TB treatment for severely ill patients has again been recommended – an illustration of the research gaps. Our data should assist the selection of parameters to be included in such screening and diagnostic algorithms in countries such as ours.

## Conclusion

From our retrospective study, differentiated WCC and CRP are not useful for screening, excluding or diagnosing TB in hospitalised patients in our setting.

## Recommendations

Moving forward, prospective data collection with a more targeted sample and a better overview of the final diagnosis would negate many of the abovementioned limitations. Inclusion of participants from primary and secondary care levels, symptom-based inclusion parameters, review of more diagnostic modalities (histology, LAM, empiric) and follow-up of those empirically diagnosed are all ways of refining future data.

## Appendix 1

**TABLE 1-A1:** Standards for Reporting of Diagnostic Accuracy Studies (STARD) checklist.

Section and topic	Number	Item	Reported on page number
**Title or abstract**	1	Identification as a study of diagnostic accuracy using at least one measure of accuracy (such as sensitivity, specificity, predictive values or AUC)	1
**Abstract**	2	Structured summary of study design, methods, results and conclusions (for specific guidance, see STARD for Abstracts)	1
**Introduction**	3	Scientific and clinical background, including the intended use and clinical role of the index test	3–4
	4	Study objectives and hypotheses	3–4
**Methods**			
Study design	5	Whether data collection was planned before the index test and reference standard were performed (prospective study) or after (retrospective study)	5
Participants	6	Eligibility criteria	5
	7	On what basis potentially eligible participants were identified (such as symptoms, results from previous tests, inclusion in registry)	5–6
	8	Where and when potentially eligible participants were identified (setting, location and dates)	5–6
	9	Whether participants formed a consecutive, random or convenience series	5
Test methods	10a	Index test, in sufficient detail to allow replication	6
	10b	Reference standard, in sufficient detail to allow replication	5
	11	Rationale for choosing the reference standard (if alternatives exist)	14
	12a	Definition of and rationale for test positivity cut-offs or result categories of the index test, distinguishing pre-specified from exploratory	P7: Mainly exploratory since potential cut-offs determined based on the ROC curves, for comparison purposes with other studies a few pre-specified cutpoints from literature used in Results section
	12b	Definition of and rationale for test positivity cut-offs or result categories of the reference standard, distinguishing pre-specified from exploratory	See comment page 6, also page 5
	13a	Whether clinical information and reference standard results were available to the performers/readers of the index test	N/A
	13b	Whether clinical information and index test results were available to the assessors of the reference standard	13
Analysis	14	Methods for estimating or comparing measures of diagnostic accuracy	7
	15	How indeterminate index test or reference standard results were handled	6
	16	How missing data on the index test and reference standard were handled	7
	17	Any analyses of variability in diagnostic accuracy, distinguishing pre-specified from exploratory	N/A (it was all exploratory re index test)
	18	Intended sample size and how it was determined	5
**Results**			
**Participants**	19	Flow of participants, using a diagram	8 (various variables used as index tests)
	20	Baseline demographic and clinical characteristics of participants	8
	21a	Distribution of severity of disease in those with the target condition	N/A
	21b	Distribution of alternative diagnoses in those without the target condition	N/A
	22	Time interval and any clinical interventions between index test and reference standard	6
**Test results**	23	Cross tabulation of the index test results (or their distribution) by the results of the reference standard	8–12
	24	Estimates of diagnostic accuracy and their precision (such as 95% confidence intervals)	8–11
	25	Any adverse events from performing the index test or the reference standard	N/A
**Discussion**	26	Study limitations, including sources of potential bias, statistical uncertainty and generalisability	13–14
	27	Implications for practice, including the intended use and clinical role of the index test	14–15
**Other information**			
	28	Registration number and name of registry	Not applicable
	29	Where the full study protocol can be accessed	17
	30	Sources of funding and other support; role of funders	17

AUC, areas under the curve; STARD, Standards for Reporting of Diagnostic Accuracy Studies; N/A, not applicable.
